# Supplementary material for: Direct Growth of Patterned Vertical Graphene Using Thermal Stress Mismatch between Barrier Layer and Substrate
Source: Nanomaterials (Basel). 2023 Mar 31;13(7):1242. doi: 10.3390/nano13071242 (PMC10097022; doi:10.3390/nano13071242)
Supplement: Supplementary file 1 [file nanomaterials-13-01242-s001.zip › nanomaterials-2281957-supplementary.pdf]

nanomaterials MDPI

Supplementary Materials

Direct Growth of Patterned Vertical Graphene Using Stress Mismatch between Sacrificial Layer and Substrate

Fengsong Qian [1,§], Jun Deng [1,§], Xiaochen Ma [1], Liangchen Hu [1], Guosheng Fu [2], and Chen Xu [1,*]

[1] Key Laboratory of Optoelectronics Technology, Ministry of Education, Beijing University of Technology, Beijing 100124, China
[2] Fert Beijing Institute and School of Microelectronics, Beihang University, Beijing 100191, China
* Correspondence: xuchen58@bjut.edu.cn
† These authors contributed equally to this work.

Table S1. Physical and mechanical properties used in the simulation of stress distribution.

	Thermal conductivity		CTE		Elastic modulus		Poisson's ratio		References
	T (°C)	λ (W/m·K)	T (°C)	α (10^{-6}/K)	T (°C)	E (GPa)	T (°C)	ν	
Si	20	139.4	27	2.59	25	168.1	27	0.278	[42-45]
	200	82.4	127	3.2	100	167.6			
	400	50.3	227	3.59	200	166.6			
	600	36.7	327	3.83	300	164.8			
	800	27.4	427	3.99	400	163.2			
	1000	23.7	527	4.1	500	162.4			
					600	160.3			
SiO_2	27	1.3	0	0.4	0	71.8	27	0.19	[46-49]
			100	0.62	100	71.4			
			200	0.75	200	70.8			
			300	0.7	300	70.5			
			400	0.65	400	70.2			
			500	0.6	500	70.1			
			600	0.5	600	70.05			
Cr	27	93	127	8.12	27	275	27	0.21	[50-53]
	127	91.8	227	9	77	274			
	227	87.7	327	9.58	127	273			
	327	83.3	427	10.1	177	272			
	427	78.8	527	10.8	227	270			
	527	74.9	627	11.3	277	268			
	627	71.8	727	11.8	327	265			
	727	69.6	827	12.2	377	262			
			927	12.7					
VG	27	2000	27	−1.1	27	49	27	0.16	[54-57]
	127	1200	100	−0.4	200	50			
	227	1010	200	0.5	400	51			
	327	900	300	1.4	600	53			
	427	690	400	2.3	800	55			
	527	600							

627	510	500	3.3	1000	61
727	500				

Nanomaterials **2022**, *5*, x. https://doi.org/10.3390/xxxxx www.mdpi.com/journal/nanomaterials

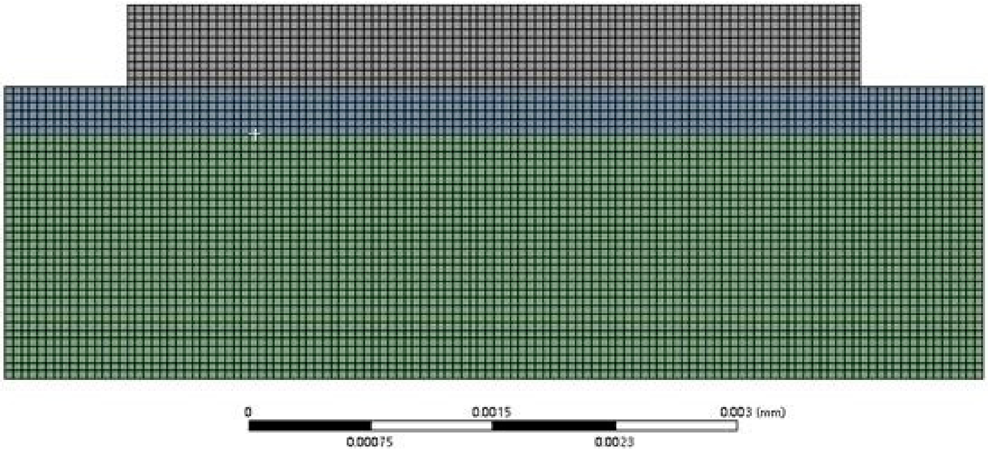

Figure S1. FE model for thermal stress analysis of a SiO_2/Si substrate with patterned Cr film (before VG synthesis).

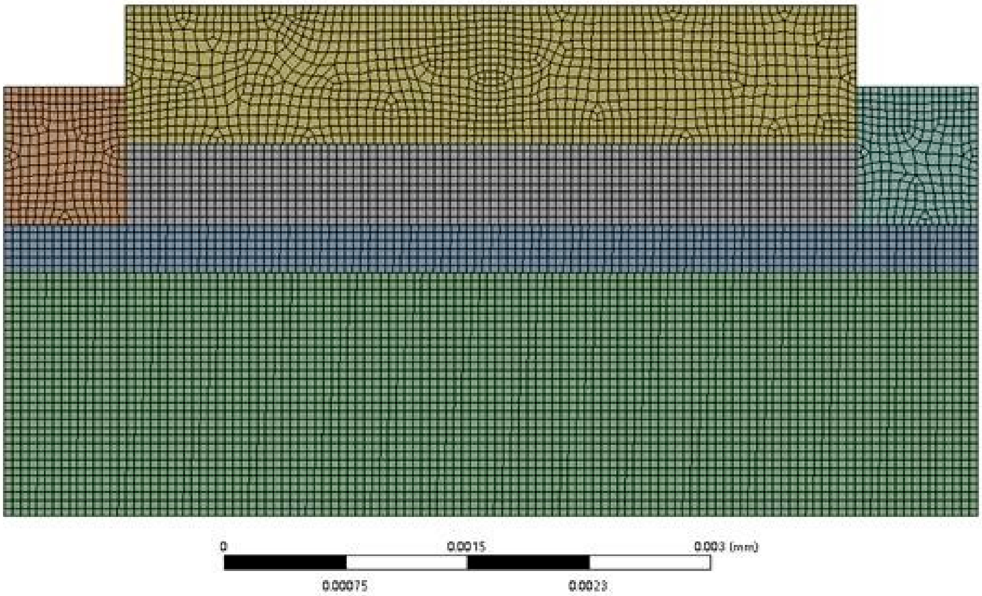

Figure S2. FE model for thermal stress analysis of a SiO_2/Si substrate with VG and patterned Cr film (after VG synthesis).

References

42. Yamasue, E.; Susa, M.; Fukuyama, H.; Nagata, K., Thermal conductivities of silicon and germanium in solid and liquid states measured by non-stationary hot wire method with silica coated probe. *J. Cryst. Growth* **2002**, *234*, 121-131.
43. Zhang, W.; Yu, H.; Lei, S.; Huang, Q., Modeling of silicon thermal expansion using strained phonon spectra. *J. Micromech. Mi-croeng.* **2012**, *22*, 85007.
44. Jeung-hyun, J.; Sung-hoon, C.; Se-Ho, L.; Dongil, K., Evaluation of elastic properties and temperature effects in Si thin films using an electrostatic microresonator. *J. Microelectromech. Syst.* **2003**, *12*, 524-530.
45. Cho, C., Characterization of Young's modulus of silicon versus temperature using a "beam deflection" method with a four-point bending fixture. *Curr. Appl. Phys.* **2009**, *9*, 538-545.
46. Yamane, T.; Nagai, N.; Katayama, S.; Todoki, M., Measurement of thermal conductivity of silicon dioxide thin films using a 3ω method. *J. Appl. Phys.* **2002**, 91, 9772-9776.

47. Li, C.; Yang, M.; Quan, B.; Guan, W., Stress Simulation Analysis of Multilayer Film Deposition. Laser & Optoelectronics Progress **2018**, *55*, 043101.
48. Soh, M. T. K.; Thurn, J.; Thomas, J. H.; Talghader, J. J., Thermally induced stress hysteresis and co-efficient of thermal expansion changes in nanoporous SiO_2. *J. Phys. D: Appl. Phys.* **2007**, *40*, 2176-2182.
49. Çetinörgü, E., A new method to experimentally determine the thermal expansion coefficient, Poisson's ratio and Young's modulus of thin films. *J. Mater. Sci.* **2009**, *44*, 2167-2170.
50. Moore, J. P.; Williams, R. K.; Graves, R. S., Thermal conductivity, electrical resistivity, and Seebeck coefficient of high-purity chromium from 280 to 1000 K. *J. Appl. Phys.* **1977**, *48*, 610-617.
51. White, G. K.; Roberts, R. B.; Fawcett, E., Thermal expansion of Cr and CrV alloys. I. Experiment. *J. Phys. F: Met. Phys.* **1986**, *16*, 449-459.
52. Choi, I.; Brandl, C.; Schwaiger, R., Thermally activated dislocation plasticity in body-centered cubic chromium studied by high-temperature nanoindentation. *Acta Mater.* **2017**, *140*, 107-115.
53. Wu, D.; Nieh, T. G., Incipient plasticity and dislocation nucleation in body-centered cubic chromium. *Mater. Sci. Eng. A* **2014**, *609*, 110-115.
54. Prasher, R., Thermal boundary resistance and thermal conductivity of multiwalled carbon nanotubes. *Phys. Rev. B* **2008**, *77*, 75424.
55. Akikubo, K.; Kurahashi, T.; Kawaguchi, S.; Tachibana, M., Thermal expansion measurements of nano-graphite using high-tem-perature X-ray diffraction. *Carbon* **2020**, *169*, 307-311.
56. Maruyama, T.; Eto, M.; Oku, T., Elastic modulus and bend strength of a nuclear graphite at high temperature. *Carbon* **1987**, *25*, 723-726.
57. Blakslee, O. L.; Proctor, D. G.; Seldin, E. J.; Spence, G. B.; Weng, T., Elastic Constants of Compression-Annealed Pyrolytic Graph-ite. *J. Appl. Phys.* **1970**, *41*, 3373-3382.
